# The iron–sulfur‐containing HypC‐HypD scaffold complex of the [NiFe]‐hydrogenase maturation machinery is an ATPase

**DOI:** 10.1002/2211-5463.12743

**Published:** 2019-10-29

**Authors:** Kerstin Nutschan, Ralph P. Golbik, R. Gary Sawers

**Affiliations:** ^1^ Institute for Biology/Microbiology Martin‐Luther University Halle‐Wittenberg Germany; ^2^ Institute of Biochemistry and Biotechnology Martin‐Luther University Halle‐Wittenberg Germany

**Keywords:** [NiFe]‐hydrogenase, HybG, HypC chaperone, HypD, iron–sulfur cluster, metalloenzyme maturation

## Abstract

HypD and HypC, or its paralogue HybG in *Escherichia coli*, form the core of the scaffold complex that synthesizes the Fe(CN)_2_CO component of the bimetallic NiFe‐cofactor of [NiFe]‐hydrogenase. We show here that purified HypC‐HypD and HybG‐HypD complexes catalyse hydrolysis of ATP to ADP (*k*_cat_ ≅ 0.85·s^−1^); the ATPase activity of the individual proteins was between 5‐ and 10‐fold lower than that of the complex. Pre‐incubation of HypD with ATP was necessary to restore full activity upon addition of HybG. The conserved Cys41 residue on HypD was essential for full ATPase activity of the complex. Together, our data suggest that HypD undergoes ATP‐dependent conformational activation to facilitate complex assembly in preparation for substrate reduction.

## Abstract 2

The iron–sulfur protein HypD and the small HypC chaperone form the core of the [NiFe]‐hydrogenase maturation machinery. We report here that both proteins have intrinsic ATP‐hydrolyzing activity and that this activity is synergistically enhanced in the HypD‐HypC complex. Our results support a model whereby HypD undergoes ATP‐dependent conformational activation, which allows manifestation of optimal ATPase activity upon HypC binding.

## Graphical abstract

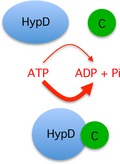

**Abbreviations**
CO: carbon monoxide
CO_2_: carbon dioxide
HEPES: (4‐(2‐hydroxyethyl)‐1‐piperazineethanesulfonic acid
Hyd: [NiFe]‐hydrogenase

The catalytically active large subunit of [NiFe]‐hydrogenases (Hyd) harbours a NiFe(CN)_2_CO‐cofactor 1. Synthesis of this cofactor requires the activities of six Hyp proteins, but not all steps catalysed by these proteins are understood. While it is known that the CN^−^ ligands are synthesized from carbamoylphosphate through the combined actions of the HypEF maturation proteins 2, 3, the metabolic origin(s) of the CO ligand remains unresolved. It is likely that two other maturation proteins, HypC and HypD, are directly involved in CO ligand synthesis. This is suggested because these proteins form a complex that has been shown to coordinate the Fe(CN)_2_CO moiety of the cofactor via conserved amino acid residues Cys 41 on HypD (*Escherichia coli* numbering) and Cys2 on HypC 4, 5, 6. Furthermore, HypD has a [4Fe–4S] cluster and is the only one of the Hyp proteins that is redox‐active. It has been hypothesized 7, 8 that electron transfer is a prerequisite for generation of CO if it originates from endogenously produced CO_2_ through the activity of a decarboxylase, as well as for cyanide transfer from the thiocyanate generated on HypE 9.

Structural analysis of the HypC‐HypD complex 10, 11 identified a conserved thioredoxin‐like fold linking the [4Fe–4S] cluster with Cys41, and results of a recent study have confirmed that HypD undergoes thiol‐disulphide exchange, which would be commensurate with a proposed 2‐electron and 2‐proton reduction/dehydration of CO_2_ to CO 1, 12. Further circumstantial evidence supporting CO_2_ as a potential source of CO for diatomic ligand biosynthesis was the finding that anaerobically isolated HypC and its paralogue, HybG in *E. coli*, showed a signal in the infrared region of the spectrum characteristic of Fe‐bound CO_2_ 8. The appearance of this signal was oxygen‐sensitive and was dependent on the conserved Cys2 residue, which is essential for maturation activity of HypC.

As well as these unresolved issues, a further barrier that would need to be overcome whether intracellularly produced CO_2_ is indeed the substrate for CO ligand synthesis is that the recently determined redox potential of the [4Fe–4S] in isolated HypD is too positive (*E* ^o^′ = −260 mV 12) to drive the reduction of CO_2_, which requires a substantially more negative redox potential (*E* ^o^′ = −530 mV). Such a thermodynamic barrier could, however, be overcome whether the electron transfer reaction within HypD was coupled to ATP hydrolysis, as for example in analogy to the role of the iron protein of nitrogenase 13. HypD is structurally unlike any other protein in the pdb database 11, and no classical ATP‐binding motif is discernible in the protein. HypD does have a Rossmann fold‐like sequence (Fig. 1A), but this is not located on the same face of the protein as the conserved and essential Cys41 residue, based on the crystal structure 10. In this study, we demonstrate not only that HypD has a low intrinsic ATPase activity, but surprisingly that HypC and its paralogue HybG also have a similar activity. Notably, however, complex formation of HypD with either HypC or HybG significantly stimulates the ATPase activity of the heterodimer, but only after ATP was first incubated with HypD.

**Figure 1:**
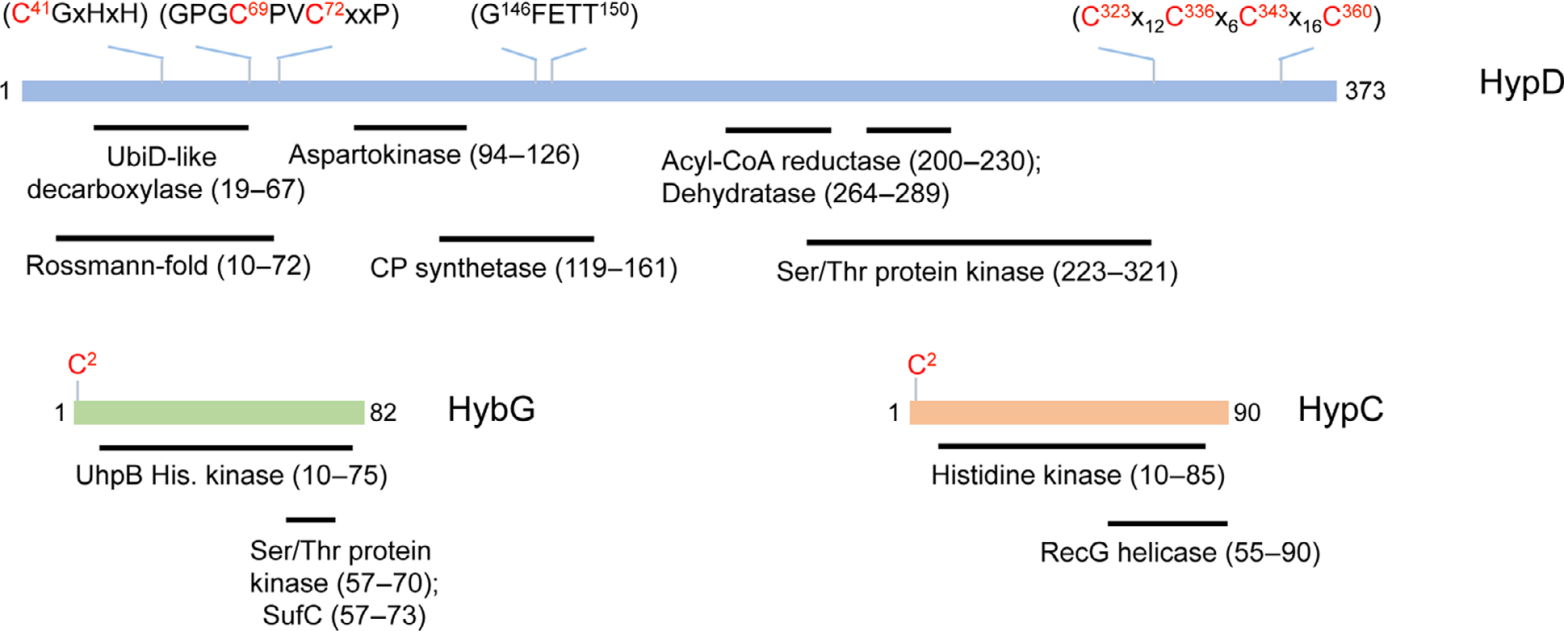
Schematic representation of key motifs in HypD, HypC and HybG. Conserved amino acid motifs in HypD (A), HybG (B) and HypC (C) are shown above the respective rectangles representing the linearized proteins. Key cysteinyl residues are shown in red. Below each protein is the region that shows amino acid similarity to various proteins based on an ExPASy comparison of known proteomes. The extent of the similarity is depicted by the black bar. For HypD, these include UbiD‐like decarboxylase from *Hyphomicrobium denitrificans* (amino acids 314–360 show 49% similarity to HypD); aspartokinase from *Dehalococcoides mccartyi* (amino acids 11–43 show 55% similarity to HypD); acyl‐CoA reductase from *Azospirillum brasilense* (amino acids 107–137 show 55% similarity to HypD); dTDP‐glucose 4,6 dehydratase from *Prevotella ruminicola* (amino acids 263–288 show 62% similarity to HypD); carbamoylphosphate synthetase from *Geobacter metallireducens* (amino acids 113–155 show 55% similarity to HypD); serine/threonine protein kinase from *Halomonas salina* (amino acids 514–612 show 45% similarity to HypD); the region from amino acid 190–280 also has similarities to various ATP‐binding proteins from *Pelagibacter ubique, Arthobacter globiformis*,* Escherichia coli*, *Bacillus subtilis* and *Bifidobacterium galicum.* HybG has similarity to UhpB histidine kinase and a serine/threonine protein kinase from *Actinomyces bovis* (amino acids 79–134 show 43% similarity to HybG) and to the SufC ATPase from *P. ruminicola* (amino acids 210–236 show 67% similarity to HybG). HypC has similarity to histidine kinase from *Cupriavidus metallireducens* (amino acids 446–515 show 40% similarity to HypC) and RecG from *Enterococcus faecalis* (amino acids 306–341 show 53% similarity to HypC).

## Materials and methods

### Bacterial strains, plasmids and growth conditions

The *E. coli* strains used included MC4100 14, and its isogenic derivative BEF314 (Δ*hypB‐E*) 15. The plasmids used included pT‐hypDEFhybG_Strep_ pIBA3hybG_WT_, pIBA3hybG_C2A_, pIBA3hypC_Strep_ 8, pT7‐7hypC_C2A_ 16, pIBA5hypD_WT_, pIBA5hypD_C41A_, pT7‐7hypD_C41A_EFhypC_Strep_ and pT7‐7hypD‐hypC_Strep_ 17. pT7‐7hypD_C41A_EFhybG_Strep_ was constructed by converting the DNA sequence at codon 41 in the *hypD* of plasmid pT7‐7hypDEFhybG_Strep_ from that coding for Cys to Ala using PCR‐based mutagenesis (Q5^®^ Site‐Directed Mutagenesis Kit procedure of New England Biolabs, Ipswich, MA, USA) and the oligonucleotides fwd: 5′‐CGG ATT ATG GAA GTG GCG GGC GGT CAT ACC CAC‐3′ and rev: 5′‐GTG GGT ATG ACC GCC CGC CAC TTC CAT ATT CCG‐3′.

*Escherichia coli* strains were transformed with the appropriate plasmid and were grown in modified TB medium 8, containing 100 μg·mL^−1^ of ampicillin at 37 °C on a rotary shaker until an optical density of 0.4 at 600 nm was reached. Note that when BEF314 was used, the growth medium included 25 μg·mL^−1^ of chloramphenicol. Gene expression was induced by the addition of 0.2 μg·mL^−1^ anhydrotetracycline followed by incubation with gentle shaking at 30 °C for 3–5 h. Cells were harvested (OD_600nm_ of 1.0) by centrifugation for 15 min at 50 000 ***g*** at 4 °C, and cell pellets were used either immediately or stored at −20 °C until use.

### Protein purification

All protein purification steps were carried out under anaerobic conditions in an anaerobic chamber (Coy Laboratories, Grass Lake, MI, USA). The HypC_strep_‐HypD complex, HypC_strep_, HybG_strep_, and HypD_strep_ were purified from BEF314, while HybG_strep_‐HypD and the HypC_strep_‐HypD complexes were purified from MC4100. Henceforth, the ‘strep’ subscript will be omitted when referring to these proteins and complexes. Strep‐tagged proteins and protein complexes were purified exactly as described 8. Purified proteins were buffer‐exchanged into 50 mm Tris/HCl pH 8 containing 150 mm NaCl, concentrated by use of Vivaspin ultra‐filters (Sartorius AG, Göttingen, Germany) with 5, 10 or 30 kDa cut‐off, as appropriate for the protein being concentrated. Purified proteins were stored at −80 °C. Determination of protein concentration was done as described 18.

### Determination of ATP hydrolysis

The ATPase activity of Hyp proteins was determined by an HPLC‐based assay. For the assay, a ZIC^®^‐HILIC column (PEEK 150 × 4.6 mm, 5 µm, 200 Å; Merck, Darmstadt, Germany) was used attached to a Hitachi Elite LaChrome HPLC system. Proteins (typically 50 μm of each) were equilibrated in 50 mm HEPES, 2 mm MgCl_2,_ pH 7.4 and incubated with 500 μm ATP in a final volume of 0.4 mL at 37 °C for 30 min. In ATP‐dependent activation reactions, 50 μm of either protein was incubated with 500 μm of ATP for 15 min at 37 °C and the second protein (also at 50 μm) was added and the incubation was continued for a further 15 min. After 30 min, 0.6 mL of 65% acetonitrile and 35% 100 mm ammonium acetate pH 4.5 were added and the mixture was centrifuged for 10 min at 15 000 x ***g*** in a desk‐top centrifuge at RT. The supernatant was filtered through a 0.2 µm filter (Sartorius), and ATP and ADP were separated using an isocratic gradient (flow rate 0.8 mL·min^−1^; 20 min; detection at 254 nm). Peak areas were integrated using the program EZchrom Elite. The amount of ADP formed was determined using a calibration curve prepared using an ADP solution of defined concentration, and the activity is presented as specific activity (μmol ATP hydrolysed·min^−1^·mg of protein^−1^). Kinetic data were investigated according to Michaelis–Menten and Eadie using the program KaleidaGraph (synergy Software, Dubai, UAE).

All experiments reported in this study were performed using minimally using three biological replicates, unless otherwise stated.

## Results

### HybG‐HypD and HypC‐HypD complexes have ATPase activity

**Figure 2:**
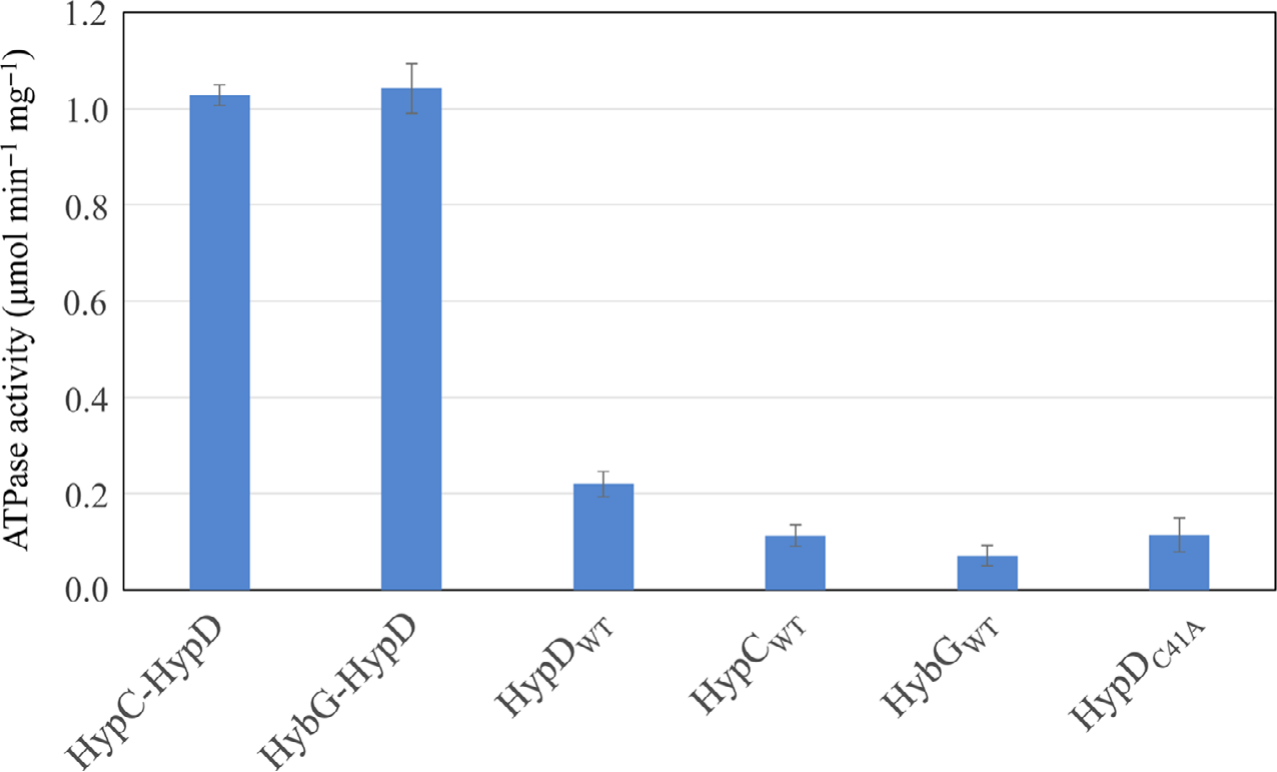
HypC‐HypD and HybG‐HypD complexes have ATPase activity. One unit of enzyme activity represents μmol of ATP hydrolysed·min^−1^. The error bars represent standard deviation.

Performing blast searches with the ExPASy web tool (web.expasy.org) using HypD, we identified short peptide sequences exhibiting amino acid sequence similarity to UbiD‐type decarboxylase, carbamoylphosphate synthetase and acyl‐CoA reductase and dehydratases from different microorganisms (Fig. 1A; see legend for % similarities and the bacterial source of each sequence). HypD also showed some amino acid similarity with kinases and an N‐terminal stretch of approximately 60 amino acids in length bore significant similarity to a Rossmann fold, as has been previously described for HypD 10, 11. Surprisingly, HypC and its paralogue HybG both exhibited amino acid sequence similarity to histidine kinases and other ATP‐dependent enzymes (Fig. 1B,C). Together, these observations are consistent with our contention that the HypC‐HypD complex might use ATP as a substrate. To test this, we analysed the ability of the isolated HypC‐HypD complex to catalyse the hydrolysis of ATP (Fig. 2). Anaerobically purified HypD‐HypC and HypD‐HybG complexes hydrolysed ATP to ADP at a rate of approximately 1 μmol·min^−1^·mg^−1^ (*k*_cat_ = 0.85 s^−1^). Small amounts of HypE, which also has an ATPase activity 9, occasionally co‐purified with the HybG‐HypD or HypC‐HypD complexes 17 when they were isolated from strain MC4100. To exclude that the ATPase activity of the HypC‐HypD complex was due to contamination with HypE, the HypC‐HypD complex was purified from strain BEF314 (Δ*hypBCDE*) 15 carrying plasmid pT7‐7hypD‐hypC_Strep_ 17 and a similar activity was measured to that shown in Fig. 2, thus excluding that the ATPase activity determined for the complex was due to HypE contamination.

**Figure 3:**
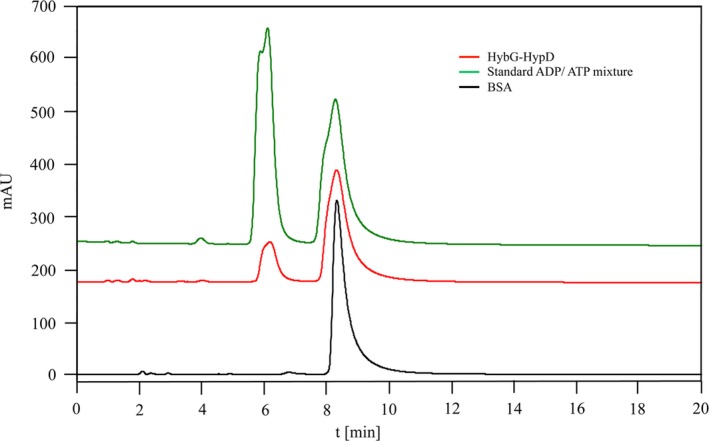
Chromatographic separation of ATP and ADP. An equimolar (0.5 mm each) mixture of ATP and ADP (green trace) was separated on a ZIC^®^‐HILIC column, as described in the Materials and methods section of the main text. The peak eluting at 6 min represents ADP and that eluting at 8.5 min represents ATP. A representative trace showing the ATPase activity of the HybG‐HypD complex after incubation under standard assay conditions is shown by the red trace, while BSA reveals no hydrolysis of ATP to ADP during the time period of the assay (black trace) and acted as a negative control.

HypD purified after over‐production in strain BEF314, and independently of HypC or HybG, had an ATP‐hydrolysing activity that was only approximately 20% that of the complex (Fig. 2). Surprisingly, both HypC and HybG also showed weak, intrinsic ATPase activities that were in the range of 0.1–0.14 μmol·min^−1^·mg^−1^ (Fig. 2). It should be noted that no chemical hydrolysis of ATP during the period of the assay was observed, nor did BSA cause hydrolysis of ATP (Fig. 3), indicating that the ATP hydrolysis catalysed by HypC, HypD and HybG was specific. Moreover, AMP was never observed as a reaction product.

### Pre‐incubation of HypD with ATP restores full ATPase activity when HypC is added

**Figure 4:**
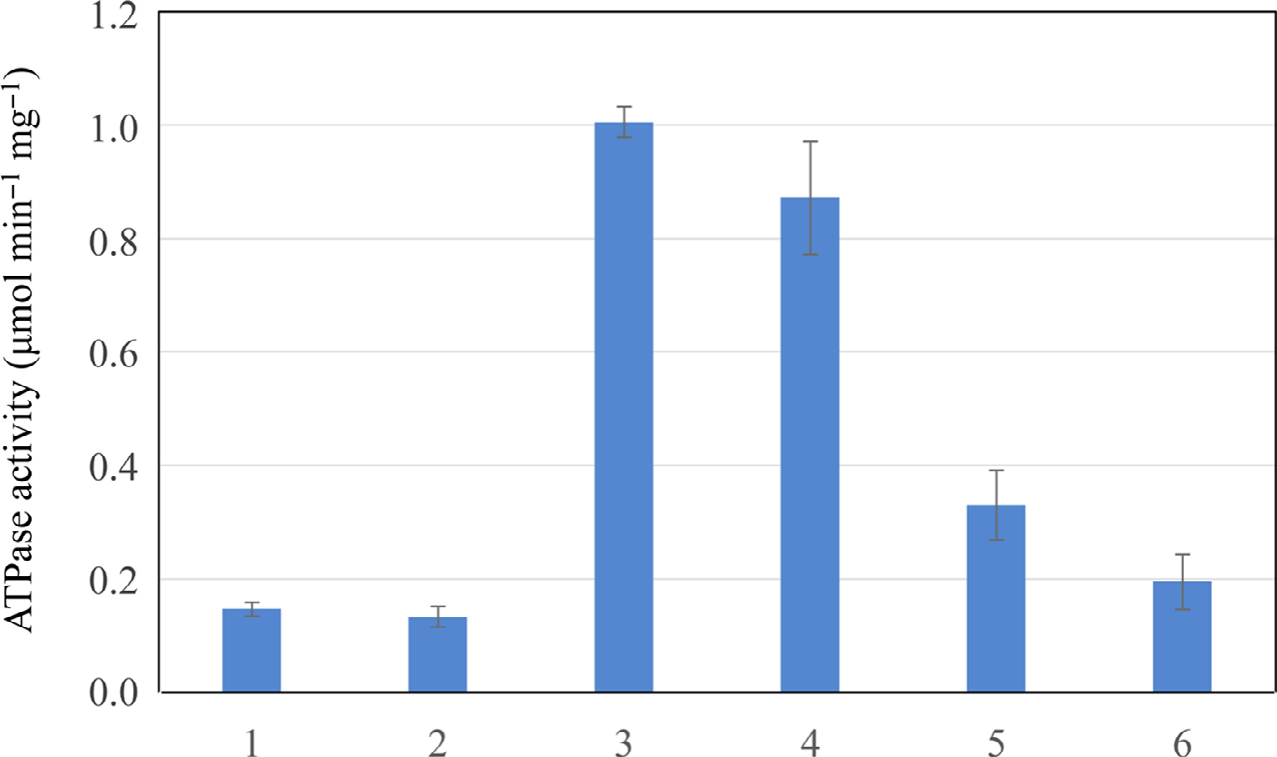
ATP‐dependent stimulation of HypD ATP‐hydrolysing activity. In the first two reactions, HypD was mixed with either HybG (reaction 1) or HypC (reaction 2) and ATP simultaneously, while in the latter 4 reactions, ATP was first incubated with HypD (reactions 3 and 4) or HybG (reaction 5) or HypC (reaction 6) for 15 min after which the second protein was added. One unit of enzyme activity represents μmol of ATP hydrolysed·min^−1^. (1) HypD + HybG + ATP; (2) HypD + HypC + ATP; (3) HypD + ATP, then HybG added after 15 min; (4) HypD + ATP, then HypC added after 15 min; (5) HybG + ATP, then HypD added after 15 min; (6) HypC + ATP, then HypD added after 15 min. The error bars represent standard deviation.

Mixing separately purified HypD with an equimolar amount of HybG, and adding ATP at the same time, failed to restore full ATPase activity to the complex during the 30‐min assay (Fig. 4). A similar observation was made when the experiment was performed with HypC instead of HybG. Pre‐incubation of HypD and HypC together for 15 min with subsequent addition of ATP also did not restore full ATPase activity, delivering an activity similar to that determined when all three components were mixed simultaneously (data not shown). However, pre‐incubation of HypD with ATP for 15 min prior to addition of HybG resulted in nearly full recovery of ATPase activity to a level similar to that measured for the as‐isolated complex (compare Figs 2 and 4). Performing this experiment by pre‐incubating HybG or HypC with ATP and then adding HypD after a 15‐min incubation failed to restore full ATPase activity to the complex (Fig. 4). Note that during the 15 min pre‐incubation, maximally 0.6% of the ATP was hydrolysed, and therefore, ATP was not limiting in this experiment.

### The conserved Cys41 residue of HypD is essential for ATPase activity of the heterodimer

**Figure 5:**
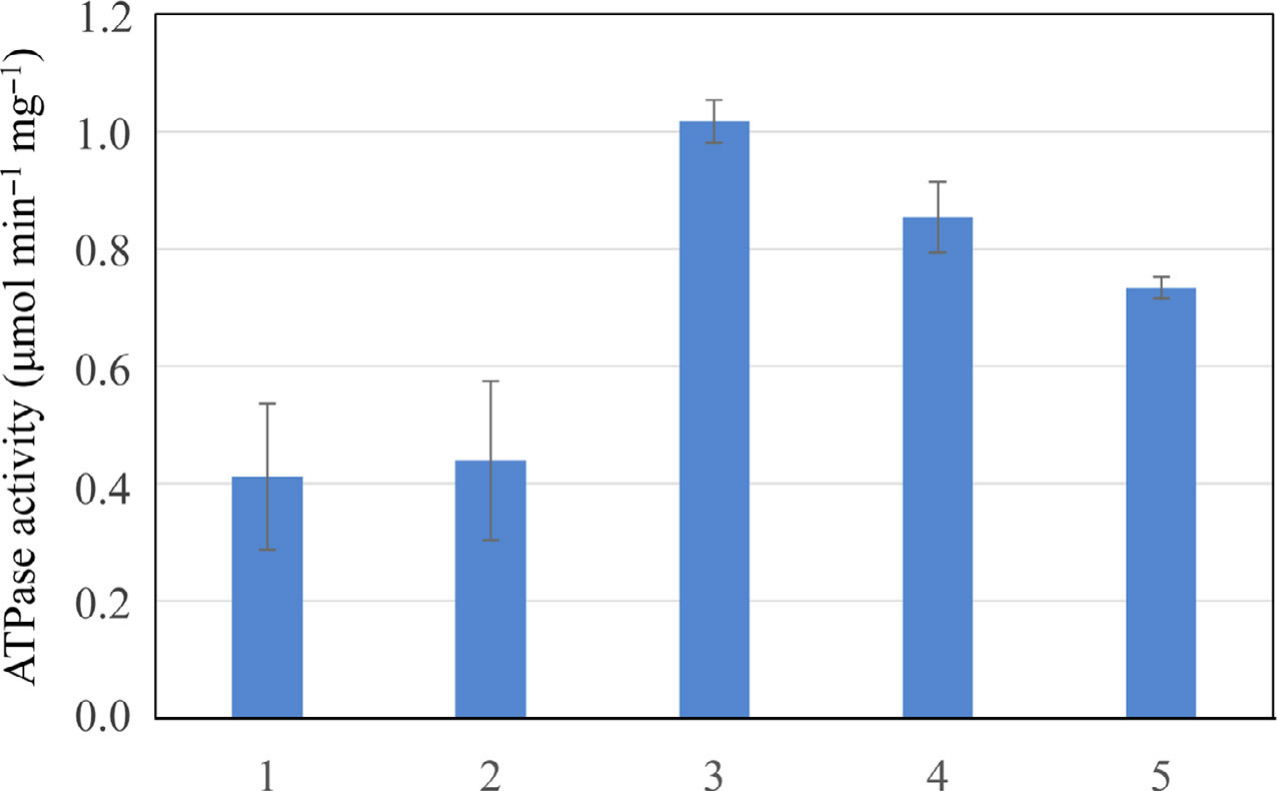
Cysteinyl residue 41 of HypD is essential for stimulation of the ATPase activity of the complex. See legend to Fig. 3 for the details of the order‐of‐addition of protein and ATP. One unit of enzyme activity represents μmol of ATP hydrolysed·min^−1^. (1) HypD_C41A_ + ATP, then HybG added after 15 min; (2) HypD_C41A_ + ATP, then HypC added after 15 min; (3) HypD + ATP, then HybG_C2A_ added after 15 min; (4) HypD + ATP, then HypC_C2A_ added after 15 min; (5) As‐isolated HypC_C2A_‐HypD + ATP. The error bars represent standard deviation.

The conserved residues Cys41 of HypD and Cys2 of HypC (and HybG) have been shown to be essential for hydrogenase maturation activity 8, 17, 19 and have been proposed to coordinate the Fe(CN)_2_CO moiety on the HypC‐HypD scaffold complex 4, 5, 6. A Cys41Ala variant of HypD (HypD_C41A_) was anaerobically purified and tested for its ability to hydrolyse ATP (Fig. 5). The results clearly show that after incubation with ATP, neither addition of HypC nor addition of HybG was capable of restoring full ATPase activity to HypD_C41A_. The HypD_C41A_ variant on its own had an ATPase activity that was approximately 70% that of native HypD when the assay was performed in the absence of HypC (Fig. 2). Together, these data indicate that the conserved Cys41 residue is essential for full ATPase activity of the complex, but not for the low intrinsic ATPase activity of the HypD protein.

To test whether the conserved Cys2 residue on the HypC family of proteins is important for the full ATPase activity of the complex, variants HypC_C2A_ and HybG_C2A_ were analysed in the ATPase assay. Wild‐type HypD was incubated with ATP, and then, either HybG_C2A_ or HypC_C2A_ was added and after a further 15‐min incubation the ATPase activities were determined (Fig. 5). The results clearly show that the Cys2 residue could be exchanged to Ala without any significant effect on the ATPase activity of the ATP‐activated complex.

### Kinetics of ATP hydrolysis

**Figure 6:**
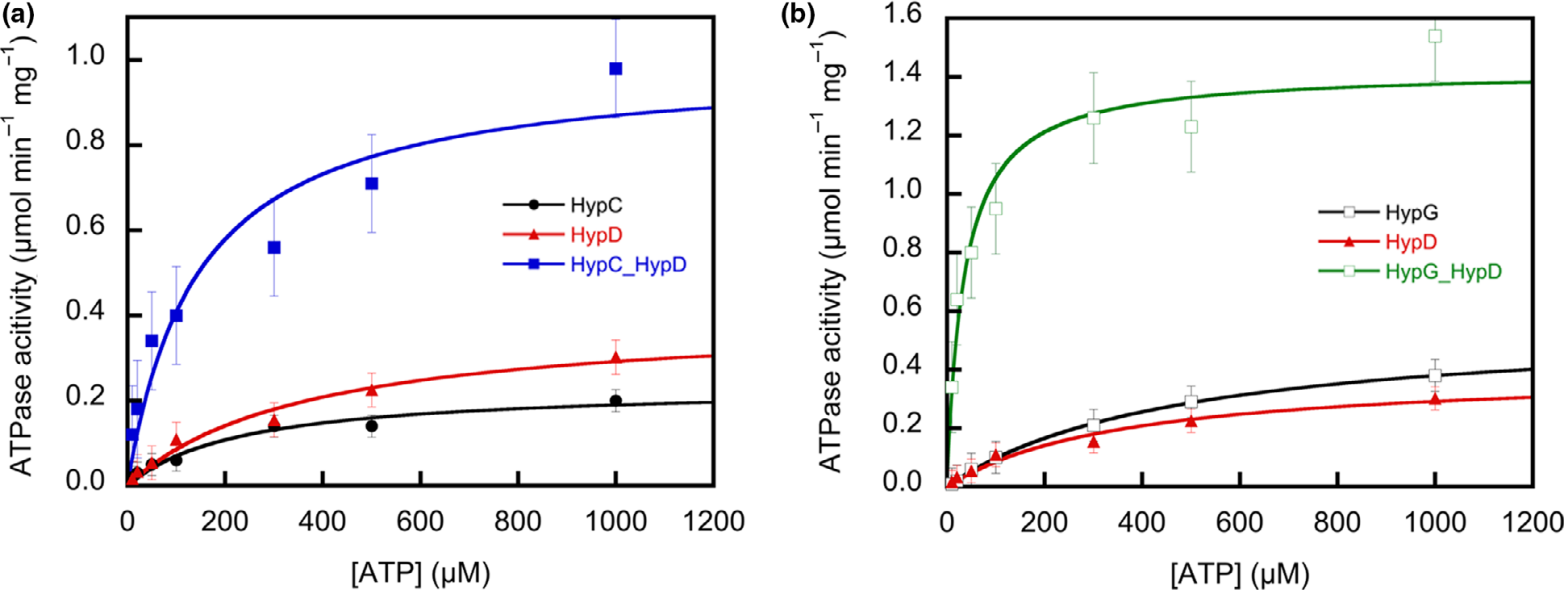
ATPase activities of the HypC, HybG, HypD and the HypC‐HypD and HybG‐HypD complexes in dependence of ATP concentration. The activity of HypD is shown as the red curve in both A and B, while the black curves shown in A and B represent the activities of HypC and HybG, respectively. The blue curve in A shows the activity of the isolated HypC‐HypD complex, while the green curve in B shows the activity of the isolated HybG‐HypD complex. These data were used as the basis to determine the kinetic data shown in Table 1 of the main text. The error bars represent standard error of the mean.

Analysis of the kinetic data (Fig. 6) according to Michaelis–Menten yielded an apparent *K*_m_ of HypD for ATP of 365 μm (Table 1). For comparison, we have included the analyses of the data according to Eadie and according to Hanes in Table 1, but will focus on the Michaelis–Menten data. While HypC showed a *K*_m_ of 235 μm, HybG had a lower affinity for ATP of *K*_m_ of 475 μm). The distribution of *V*_max_ corresponded roughly with that of the *K*_m_ values, whereby HybG had a *V*_max_ that was more than double that of HypC (Table 1). The *K*_m_ for ATP was reduced approximately 10‐fold for the HybG‐HypD complex compared with that for HypD alone, while that of the HypC‐HypD complex was ~ 50% lower than that of HypD (Table 1). The *V*_max_ values of both complexes were significantly higher than those of the individual proteins.

**Table 1:** Kinetic analysis of the enzyme data.

Protein	Michaelis–Menten^a^		Eadie^a^		Hanes^b^	
	*V*_m_ (µmol·min^−1^·mg^−1^)	*K*_m_ (µmol·L^−1^)	*V*_m_ (µmol·min^−1^·mg^−1^)	*K*_m_ (µmol·L^−1^)	*V*_m_ (µmol·min^−1^·mg^−1^)	*K*_m_ (µmol·L^−1^)
HypC	0.234 ± 0.026	235.72 ± 71.15	0.167 ± 0.025	87.61 ± 23.32	0.223	181.52
HypD	0.398 ± 0.046	364.48 ± 100.16	0.295 ± 0.035	172.31 ± 31.88	0.363	261.30
HybG	0.560 ± 0.016	475.67 ± 29.91	0.541 ± 0.067	426.38 ± 73.62	0.577	510.36
HypC‐HypD	0.995 ± 0.119	143.48 ± 55.10	0.803 ± 0.090	64.57 ± 13.92	1.041	141.11
HybG‐HypD	1.423 ± 0.082	34.78 ± 8.77	1.365 ± 0.089	28.02 ± 4.65	1.561	56.62

a Kinetic data of the v/S‐plots were investigated using the Program KaleidaGraph™ (synergy Software). Standard errors of the fitting routine are indicated for data investigation according to Michaelis–Menten and after linearization according to Eadie.

b The standard errors of enzymatic parameters derived from data investigation after linearization according to Hanes by using the laws of error propagation would exceed reasonable values and are not indicated in the table.

Incubation of the HybG‐HypD complex with equimolar concentrations (500 μm) of ATP and GTP did not cause any inhibition of ATPase activity, ruling out that GTP is a substrate for the complex. Moreover, incubation of the HybG‐HypD complex with a 50‐fold molar excess of ADP over ATP also failed to result in inhibition of ATPase activity, indicating that ADP is not a competitive inhibitor of the reaction (data not shown).

## Discussion

The discovery that the HypC‐HypD complex has an ATP‐hydrolysing activity extends to three, including those catalysed by HypF and HypE, the number of ATP‐dependent steps during the biosynthesis of the Fe(CN)_2_CO moiety of the NiFe‐cofactor. The identification of an ATPase activity for the complex would be in accord with the hypothesized involvement of the complex in the reduction of CO_2_ to generate CO 7, 8. Furthermore, the fact that full ATPase activity was observed only by the complex underscores CO_2_ as a putative substrate for the reaction because HypC has been shown to carry, and thus likely deliver to HypD, iron and CO_2_ 8. The amino acid sequence similarities identified between HypD and short peptide sequences of decarboxylases, carbamoylphosphate synthetase and a CoA reductase are also in accord with a proposed function of HypD in using CO_2_ as a substrate (as well as separately transferring a cyanyl group from HypE 1, 9) and catalysing its 2 e^−^ + 2 H^+^ reduction (formally a dehydration) to CO. Hydrolysis of ATP might therefore provide the energy required to overcome the thermodynamic barrier presented by the redox potential of HypD’s [4Fe–4S] cluster, which is insufficiently negative to reduce CO_2_ spontaneously, if CO_2_ indeed proves to be the substrate for CO diatomic ligand synthesis. However, we have not yet observed any influence of CO_2_ addition on the ATP‐hydrolysing activity of the HypC‐HypD complex (data not shown), which might be due to the fact that CO_2_ is either bound to a protein or in an iron‐bound form, as has been previously suggested 8. Another possibility is that ATP hydrolysis might be important in establishing complex formation with other components (e.g. HypE and HypF) of the maturation machinery. The fact that the HypC‐HypD complex isolated from the BEF314 (Δ*hypB‐E*) has the same ATPase activity as that isolated from a *hyp* ^+^ wild‐type strain suggests that ATP hydrolysis is probably not involved in transfer of the Fe(CN)_2_CO group from the complex to the [NiFe]‐hydrogenase large subunit. Nevertheless, future experiments can now be designed to test these possibilities.

We can rule out that ATP triggers the disassembly of the HypC‐HypD complex because we could never observe HypC being released from the as‐isolated complex upon incubation with ATP (data not shown). Rather, the observed ATP‐dependent activation of HypD, which restored full ATPase activity when either HypC or HybG was added to the mixture, suggests that an ATP‐dependent conformational activation of HypD might occur, which would promote interaction with HypC or HybG. Evidence that a conformational change in HypD occurs upon complex formation with HypC was provided by the structural analyses performed on HypD, HypC and the complex of the two proteins isolated from the archaeon *Thermococcus kodakarensis* 10, 11.

The fact that the simultaneous mixing and incubation of HypD, HypC and ATP failed to manifest the full ATPase activity suggests that the presence of HypC in solution sterically hinders the binding of ATP to HypD. HypD lacks either Walker A or B motifs 19, or a PurM‐like ATP‐binding motif, which is present in HypE 10, and thus, it is unclear what the ATP‐binding site in HypD looks like. Together with the N‐terminal motif that includes the essential C41 residue, there are two other highly conserved motifs in HypD, namely the GPGCPVCxxP (amino acids 66–75) and GFETT (amino acids 146–150) motifs (Fig. 1A), which might be considered as candidates for ATP interaction. Apart from the C41A variant, which we show in this study to lack full ATPase activity, exchanges of the conserved C69 and C72 residues of HypD also abolish both maturation activity *in vivo* and interaction with HypC *in vitro* 20. This suggests that the N‐terminal region of HypD might be important for binding ATP. It was initially rather unexpected to discover that both HypC and HybG also have a low intrinsic ATPase activity. This is likely to be of relevance, however, for the ATP‐binding site within the complex and perhaps suggests that the interface of the HypC and HypD proteins forms this site. Indeed, the structure of the HypC‐HypD complex, as well as revealing considerable conformational restraint, also revealed the formation of a deep cleft along the interface, suggestive of a substrate‐binding site 11.

Finally, it is also conceivable that ATP‐binding and hydrolysis might influence the redox chemistry of the conserved [4Fe–4S] cluster in HypD, facilitating energization of electrons necessary for the otherwise endothermic substrate reduction to produce the diatomic CO ligand. As such, the HypC‐HypD two‐component ATPase is formally similar to a class of enzymes that performs ATP‐driven redox reactions, in which the first component is an ATPase with an iron–sulfur cluster and the second component is a metalloenzyme, which is the electron acceptor and site of substrate reduction 21. Due to the fact that the HypC protein (second component) has been shown to bind iron and CO_2_ 8, coupling the ATP‐dependent conformational activation of HypD (first component) might trigger electron transfer and drive substrate reduction. Whether that substrate is indeed CO_2_ can now be addressed in future studies that incorporate ATP in the assay.

## Conflict of interest

The authors declare no conflict of interest.

## Author contributions

KN carried out the experiments. KN, RPG and GRS designed the experiments and analysed the data. GRS drafted the manuscript and conceived the study. All authors read and approved the final manuscript.
